# ALKBH5 regulates cardiomyocyte proliferation and heart regeneration by demethylating the mRNA of YTHDF1

**DOI:** 10.7150/thno.47354

**Published:** 2021-01-01

**Authors:** Zhenbo Han, Xiuxiu Wang, Zihang Xu, Yang Cao, Rui Gong, Yang Yu, Ying Yu, Xiaofei Guo, Shenzhen Liu, Meixi Yu, Wenya Ma, Yiming Zhao, Juan Xu, Xingda Li, Shuainan Li, Yan Xu, Ruijie Song, Binbin Xu, Fan Yang, Djibril Bamba, Natalia Sukhareva, Hong Lei, Manqi Gao, Wenwen Zhang, Naufal Zagidullin, Ying Zhang, Baofeng Yang, Zhenwei Pan, Benzhi Cai

**Affiliations:** 1 Department of Pharmacy at The Second Affiliated Hospital, and Department of Pharmacology (The Key Laboratory of Cardiovascular Medicine Research, Ministry of Education) at College of Pharmacy, Harbin Medical University, Harbin 150086, China; 2 Department of Clinical Pharmacology (the Heilongjiang Key Laboratory of Drug Research), Harbin Medical University, Harbin 150086, China; 3 Department of Bioinformatics, Harbin Medical University, Harbin 150086, China; 4 Department of Internal Diseases, Bashkir State Medical University, Ufa 450008, Russia

**Keywords:** Heart regeneration, cardiomyocyte proliferation, m^6^A, ALKBH5, myocardial infarction

## Abstract

N^6^-methyladenosine (m^6^A) RNA modification, a dynamic and reversible process, is essential for tissue development and pathogenesis. However, the potential involvement of m^6^A in the regulation of cardiomyocyte (CM) proliferation and cardiac regeneration remains unclear. In this study, we aimed to investigate the essential role of m^6^A modification in heart regeneration during postnatal and adult injury.

**Methods and results:** In this study, we identified the downregulation of m^6^A demethylase ALKBH5, an m6A “eraser” that is responsible for increased m^6^A methylation, in the heart after birth. Notably, *ALKBH5* knockout mice exhibited decreased cardiac regenerative ability and heart function after neonatal apex resection. Conversely, forced expression of ALKBH5 via adeno-associated virus-9 (AAV9) delivery markedly reduced the infarct size, restored cardiac function and promoted CM proliferation after myocardial infarction in juvenile (7 days old) and adult (8-weeks old) mice. Mechanistically, ALKBH5-mediated m^6^A demethylation improved the mRNA stability of YTH N^6^-methyladenosine RNA-binding protein 1 (YTHDF1), thereby increasing its expression, which consequently promoted the translation of Yes-associated protein (YAP). The modulation of ALKBH5 and YTHDF1 expression in human induced pluripotent stem cell-derived cardiomyocytes consistently yielded similar results.

**Conclusion:** Taken together, our findings highlight the vital role of the ALKBH5-m^6^A-YTHDF1-YAP axis in the regulation of CMs to re-enter the cell cycle. This finding suggests a novel potential therapeutic strategy for cardiac regeneration.

## Introduction

Heart diseases such as myocardial ischemia and heart failure are usually accompanied by an irreversible loss of cardiomyocytes (CMs). Mammalian CMs possess the capacity to regenerate only during the prenatal period or within a few days after birth 1-4. In recent years, momentous advances have been reported in this field. Most notably, Yes-associated protein (YAP), a downstream effector in the Hippo signaling pathway, has been identified as a core factor in CM proliferation 5, 6. Moreover, numerous transcription factors such as Meis1, Tbx20, and GATA4 have been identified as regulators of cardiac regeneration 7-10. The oxygen level in the heart is another critical regulator of CM proliferation after ischemic injury 11. The previous study also showed the differential expression of long non-coding RNAs in postnatal and adult murine CMs, was able to regulate CM proliferation and cardiac regeneration 12. However, the molecular mechanisms of cardiomyocyte cell cycle control remain largely unknown, and further targets for cardiac regeneration need to be explored.

N^6^-methyladenosine (m^6^A) modification is the most common modification of mammalian messenger RNAs. The process of m^6^A modification is reversible and dynamic. This modification is initiated by methyltransferase complexes (“writers”), which consist of Wilms tumor 1 associated protein (WTAP), methyltransferase-like 3 (METTL3), and methyltransferase-like 14 (METTL14), and is removed by “erasers”, which consist of fat-mass and obesity-associated protein (FTO) and α-ketoglutarate-dependent dioxygenase alkB homolog 5 (ALKBH5). In addition, m^6^A marks can be recognized by m^6^A binding proteins (“readers”), including YTHDF1-3, IGF2BP1-3, and YTHDC1-2. M^6^A modification regulates the fates of mRNAs mainly by modulating splicing, transport, stability, and nuclear localization. In recent years, numerous studies have associated dysregulated m^6^A modification with cancer development, stem cell fates, and many other biological processes. Nevertheless, to our knowledge, it remains unclear whether m^6^A modification regulates cardiomyocyte proliferation.

In this study, we revealed a marked downregulation of ALKBH5, a m^6^A eraser, after birth. Moreover, we found that the induced postnatal expression of ALKBH5 enhanced mitosis in isolated primary CMs and human induced pluripotent stem cell-derived CMs (hiPS-CMs) and improved cardiac function after heart injury. Mechanistically, ALKBH5-mediated m^6^A modification increased the expression of YTHDF1 by modulating the stability of the corresponding mRNA, thus promoting the translation of YAP, a core regulator of heart regeneration. Our findings reveal the critical role of m^6^A modification in cardiac regeneration and demonstrate the collaboration between m^6^A erasers and readers.

## Results

### ALKBH5 in neonatal heart tissue regulates cardiomyocyte proliferation

**Figure 1:**
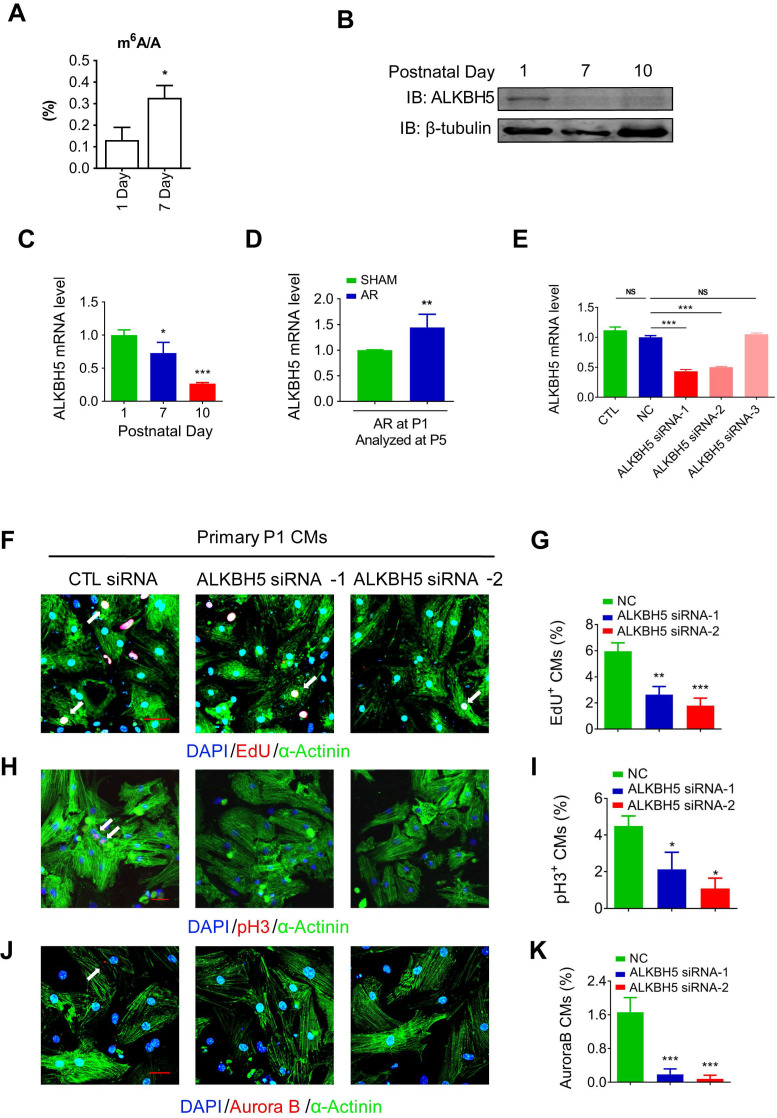
**Knock down of ALKBH5 inhibits cardiomyocyte proliferation.** (A) m6A ELISA assay of mRNA isolated from P1 and P7 hearts. *P < 0.05, n = 3. (B) Western blot assay of ALKBH5 in hearts from P1, P7 and P10 mice. β-TUBULIN was used as a loading control. (C) RT-qPCR analysis of ALKBH5 in hearts from P1, P7 and P10 mice *P < 0.05, and ***P < 0.001 (n = 4). (D) RT-qPCR analysis of ALKBH5 of hearts harvested 4 days after an AR operation on a 1-day-old mice **p < 0.01 (n = 4). (E) RT-qPCR analysis of ALKBH5 in cultured cardiomyocytes transfected with three independent ALKBH5 siRNA and negative control siRNA (NC). ***p < 0.001 (n = 4). (F-K) Cardiomyocytes isolated from P1 mice were transfected with CTL-siRNA or ALKBH5-siRNA and immunostained against EdU, phospho-histone H3 (pH3), Aurora B kinase and α-actinin (marks cardiomyocytes). DAPI was used for nuclear staining. *P < 0.05, **P < 0.01, ***P < 0.001. n = 5 per group. The arrows point to EdU/pH3/Aurora B kinase-positive signal. Scale bar, 30 μm.

To define the role of m^6^A modification in cardiomyocyte proliferation. We performed m^6^A dot blot analysis and m^6^A quantification analysis of messenger RNAs in P1 and P7 mouse hearts and found that m^6^A methylation was significantly increased after birth (Figure 1A and 1B). Next, we observed a gradual decrease in the mRNA and protein expression of ALKBH5 in cardiac tissue postnatally (Figure 1B-C). However, the expression of another m^6^A demethylase FTO, did not change after birth (Figure S1B). In addition, the mRNA and protein levels of ALKBH5 was significantly increased 4 days after apical resection injury of P1 neonatal mice (Figure 1D and Figure S1C). The expression of ALKBH5 in P1 cardiomyocytes was significantly higher than in cardiac fibroblasts (Figure S1D). These results implying that ALKBH5-mediated m^6^A modification may play a regulatory role in CM proliferation. To test this possibility, we used three independent siRNAs to silence ALKBH5 in P1 neonatal cardiomyocytes. Transfection of ALKBH5 siRNA-1 and siRNA-2 significantly reduced the expression of ALKBH5 while enhancing the level of m^6^A modification in the mRNA (Figure 1E, and Figure S1E). We then stained CMs with cell cycle markers 5-ethynyl-2'-deoxyuridine (EdU), histone H3 phosphorylated at serine 10 (pH3), and Aurora B kinase. Notably, the silencing of ALKBH5 significantly reduced the percentages of P1 CMs expressing these markers (Figure 1F-K). ALKBH5 knockdown was associated with decreases in the expression of cell-cycle activators (CDK1, CDK4, Cyclin B1 and Cyclin D1) in cultured P1 CMs (Fig. S1F). The silencing of ALKBH5 was able to decrease the cardiomyocyte number (Figure S1G). In addition, the expression of proliferation associated gene CTNND1 was significantly downregulated after ALKBH5 knockdown (Figure S1H).

**Figure 2:**
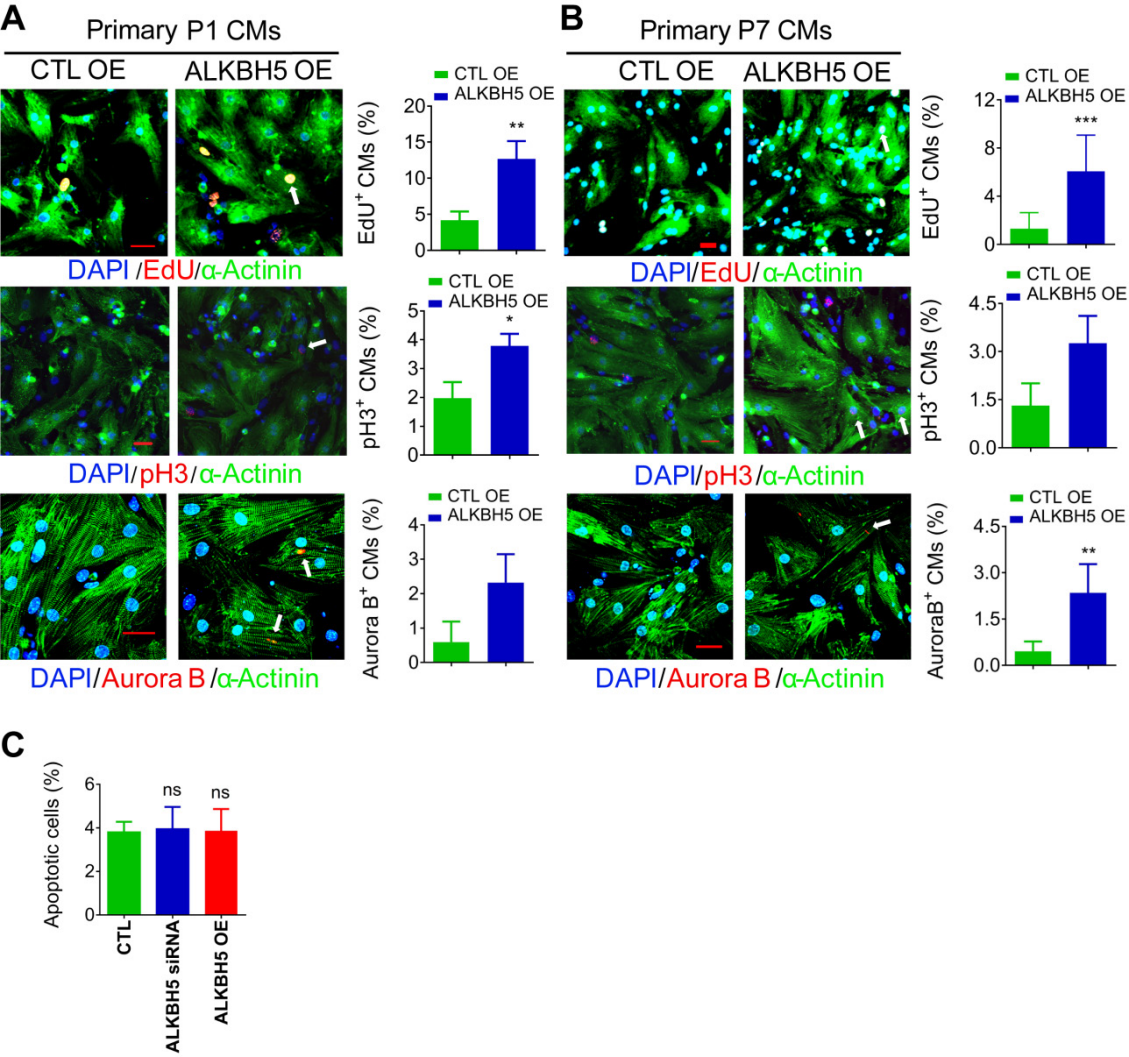
**Overexpression of ALKBH5 promotes cardiomyocyte proliferation.** (A) Neonatal P1 cardiomyocytes were transfected with CTL-plasmids or ALKBH5-overexpression plasmids for 48 hr. CMs were immunostained against EdU, phospho-histone H3 (pH3), Aurora B kinase and α-actinin (marks cardiomyocytes). DAPI was used for nuclear staining. n = 5 per group. *P < 0.05, **P < 0.01, ***P < 0.001. The arrows point to EdU/pH3/Aurora B kinase-positive signal. Scale bar, 30 μm. (B) Isolated P7 cardiomyocytes were transfected with CTL-plasmids or ALKBH5-overexpression plasmids for 48 hr. CMs were immunostained against EdU, phospho-histone H3 (pH3), Aurora B kinase and α-actinin (marks cardiomyocytes). DAPI was used for nuclear staining. n = 5 per group. *P < 0.05, **P < 0.01, ***P < 0.001. The arrows point to EdU/pH3/Aurora B kinase-positive signal. Scale bar, 30 μm. (C) The effect of ALKBH5 on apoptosis of P1 cardiomyocytes as determined by TUNEL staining (n = 4).

To further verify the role of ALKBH5 in CM proliferation, we overexpressed ALKBH5 in P1 and P7 CMs isolated from mouse hearts (Figure S1I). As expected, exogenous ALKBH5 expression led to a remarkable increase in the percentages of cell cycle marker expression in cultured P1 and P7 CMs (Figure 2A-B). Tunel assay showed that ALKBH5 KD or OE did not affect the level of apoptotic cells (Figure 2C). Taken together, these data indicate that ALKBH5-mediated m^6^A demethylation plays a regulatory role in CM proliferation.

### Deletion of ALKBH5 inhibits the regenerative capacity of neonatal hearts

**Figure 3:**
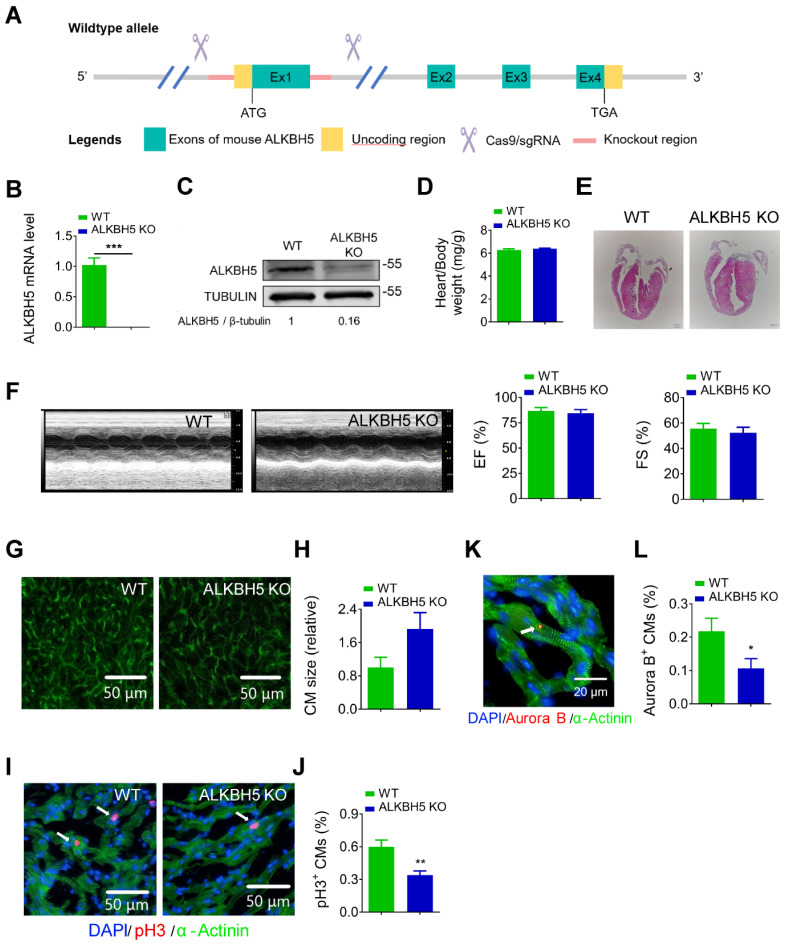
** Deletion of ALKBH5 inhibits cardiomyocyte proliferation in vivo.** (A) Schematic for generating transgenic ALKBH5 KO mice. (B) RT-qPCR analysis of ALKBH5 in hearts from P21 WT and ALKBH5 KO mice (***P < 0.001, n = 4). (C) Western-blot analysis of ALKBH5 in hearts from P21 WT and ALKBH5 KO mice. β-TUBULIN was used as a loading control. (D) Heart weight to body weight ratio of ALKBH5 KO mice and wild-type (WT) mice (n = 5). (E) HE staining of heart from 1 month-old ALKBH5 KO mice and wild-type (WT) mice. (F) Cardiac function analyzed by echocardiography. EF, ejection fraction FS, fraction shortening. (G-H) Wheat germ agglutinin (WGA) staining and quantification of P21 ALKBH5 KO (n = 8) and wild-type (WT) (n = 6) hearts. Scale bars, 50 µm. (I, J) pH3 immunofluorescence staining in P21 ALKBH5 KO and wild-type (WT) hearts and quantification of pH3-positive CMs (22587 CMs in the WT group and 10397 CMs in the ALKBH5 KO group). **P < 0.01. (K, L) Aurora B kinase immunofluorescence staining in P21 ALKBH5 KO and wild-type (WT) hearts and quantification of pH3-positive CMs (13844 CMs in the WT group and 16411 CMs in the ALKBH5 KO group). *P < 0.05. Scale bar, 20 µm.

To further study the role of ALKBH5 in cardiac regeneration, we used the CRISPR/Cas9 technique to generate *ALKBH5* knockout (KO) mice (Figure 3A). We confirmed the losses of ALKBH5 mRNA and protein expression in P21 KO mice (Figure 3B-C). There were no differences in heart size, heart/body weight ratio, or cardiac function between the *ALKBH5* KO mice and WT mice (Figure 3D-F). Notably, wheat germ agglutinin staining revealed an increase in the cross-sectional areas of CMs in hearts from *ALKBH5* KO mice (Figure 3G-H). Drastic decreases in CM mitosis and cytokinesis were observed in *ALKBH5* KO mice, as indicated by pH3 and Aurora B kinase staining (Figure 3I-L). Consistently, *ALKBH5* KO mice exhibited downregulated expression of cell-cycle activators (CDK1, CDK4, Cyclin B1, and Cyclin D1) and upregulated expression of cell cycle inhibitors (P21, P27 and meis1) (Figure S1J).

**Figure 4:**
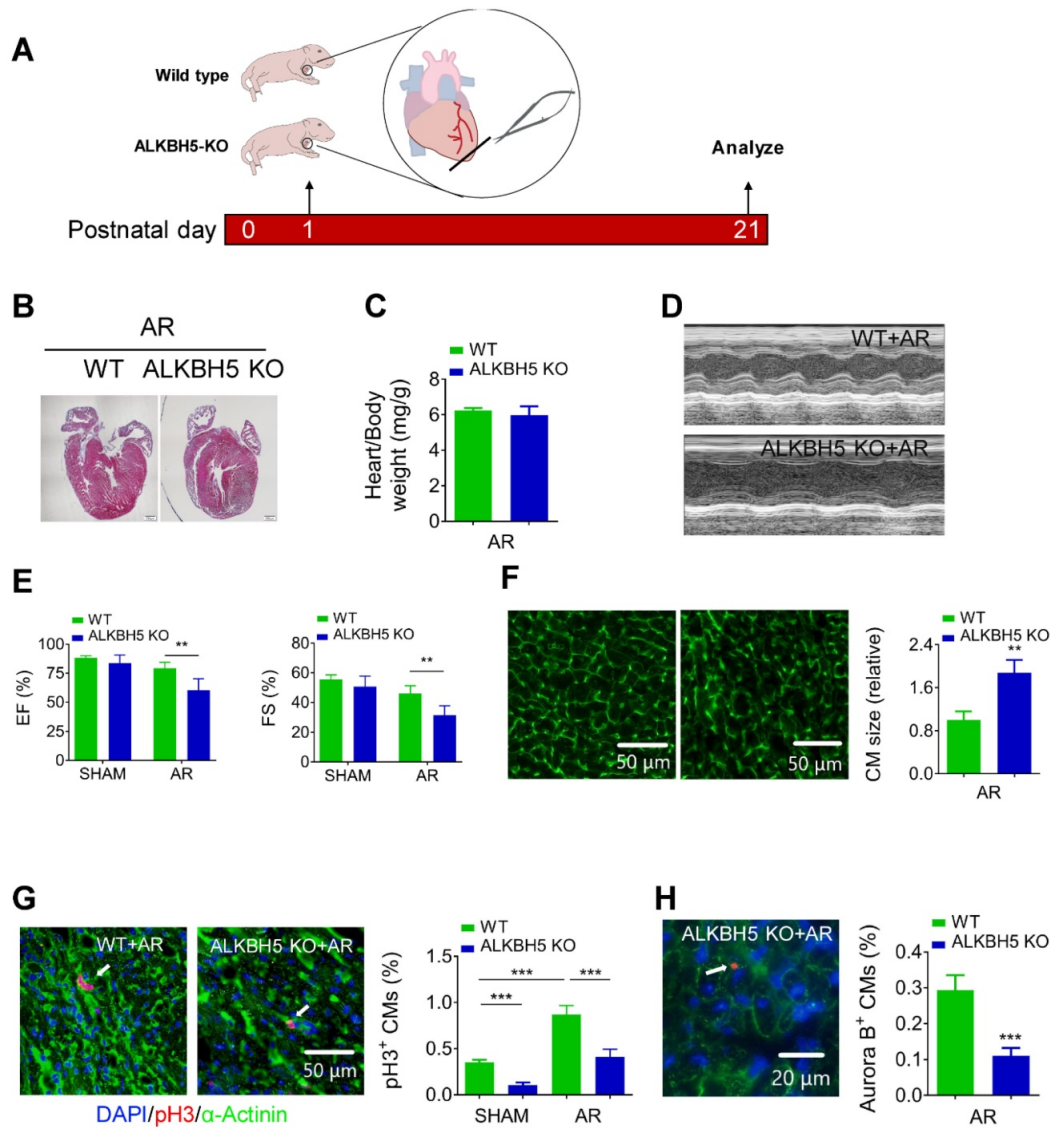
** Deletion of ALKBH5 inhibits cardiomyocyte proliferation and cardiac regeneration after apex resection (AR) injury.** (A) Schematic diagram for experimental procedure in ALKBH5 KO and wild-type (WT) mice. (B) HE staining of heart from P21 ALKBH5 KO mice and wild-type (WT) mice after treated with AR at P1. (C) Heart weight to body weight ratio of ALKBH5 KO mice and wild-type (WT) mice (n = 4). (D, E) Cardiac function analyzed by echocardiography. n = 6. **P < 0.01. (F) Wheat germ agglutinin (WGA) staining and quantification of P21 ALKBH5 KO (n = 13) and wild-type (WT) (n = 8) hearts after treated with AR at P1. **P < 0.01. Scale bars, 50 µm. (G) pH3 immunofluorescence staining in P21 ALKBH5 KO and wild-type (WT) hearts and quantification of pH3-positive CMs (9941 CMs in the AR-WT group and 6930 CMs in the AR-ALKBH5 KO group). ***P < 0.001. Scale bar, 50 µm. (H) Aurora B kinase immunofluorescence staining in P21 ALKBH5 KO and wild-type (WT) hearts and quantification of pH3-positive CMs (14038 CMs in the AR-WT group and 11287 CMs in the AR-ALKBH5 KO group). ***P < 0.001. Scale bar, 20 µm.

Next, we examined the regenerative capacities of P1 WT and ALKBH5 KO mice after apex resection (AR) injury (Figure 4A). Using HE staining, we determined the morphology of hearts and found that the injured hearts of *ALKBH5* KO mice were incapable of repair (Figure 4B). In addition, no obvious differences in heart/body weight ratio were observed between *ALKBH5* KO mice and WT mice (Figure 4C). Ejection fraction and fractional shortening analyses revealed noticeable decreases in cardiac function of *ALKBH5* KO compared to WT mice in the AR group (Figure 4D-E).

As expected, significant increases in mitosis of CMs were observed in WT AR-treated mice compared to the WT control, whereas *ALKBH5* KO led to dramatic decreases in the proliferative capacities of CMs in both SHAM- and AR-treated mice (Figure 4G). Consistently, the CM surface size was larger in the cardiac tissues of *ALKBH5* KO mice compared to those of WT controls after AR (Figure 4F). Aurora B kinase staining also confirmed a decrease in proliferative CMs in *ALKBH5* KO hearts (Figure 4H). Together, these data indicate that *ALKBH5* deletion contributes to a reduction in the regenerative capacity of the neonatal heart.

### Overexpression of ALKBH5 promotes cardiomyocyte proliferation and heart function after myocardial infarction

**Figure 5:**
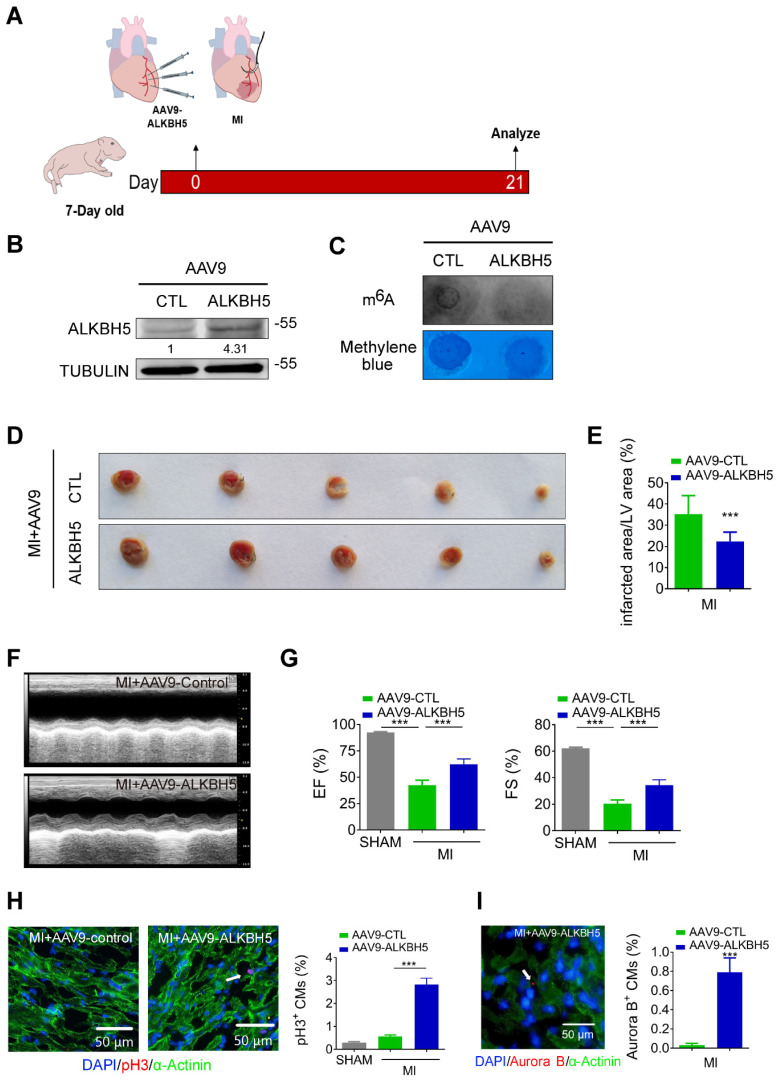
** Overexpression of ALKBH5 promotes heart regeneration of juvenile mice (7-day old) after MI.** (A) Schematic diagram for experimental procedure. (B) Western blot analysis of ALKBH5 in in hearts from P28 AAV9-ALKBH5 mice and AAV9-control mice after injected with virus at P7. (C) m^6^A dot blot assay of mRNAs in hearts from P28 AAV9-ALKBH5 mice and AAV9-control mice after injected with virus at P7. (D, E) Infarct size of hearts from MI+AAV9-ALKBH5 and MI+AAV9-control mice by 2,3,5-triphenyltetrazolium chloride (TTC) staining. ***P < 0.001. (F, G) Cardiac function of P28 AAV9-ALKBH5 mice and AAV9-control mice after treated with MI at P7. n = 5 for SHAM and MI+AAV9-CTL mice, n = 6 for MI+AAV9-ALKBH5 mice. ***P < 0.001. (H) pH3 immunofluorescence staining in P28 SHAM, MI+AAV9-ALKBH5 mice and MI+AAV9-control hearts and quantification of pH3-positive CMs (30970 CMs in the SHAM group, 7893 CMs in the MI+AAV9-control group and 16134 CMs in the MI+AAV9-ALKBH5 group). ***P < 0.001. (I) Aurora B kinase immunofluorescence staining in P28 AAV9-ALKBH5 mice and AAV9-control hearts and quantification of pH3-positive CMs (9266 CMs in the AAV9-control group and 9549 CMs in the AAV9-ALKBH5 group). ***P < 0.001.

To verify the effect of ALKBH5 overexpression on cardiac regeneration after MI, we used an AAV9-mediated delivery system to enforce the expression of ALKBH5 in mouse hearts at P4 and subjected the mice to MI at P7 (Figure 5A). The levels of ALKBH5 mRNA and protein in heart tissues increased significantly while m^6^A modification of mRNAs were decreased after AAV9-ALKBH5 injection (Figure 5B-C and Figure S1K). The 2,3,5-Triphenyltetrazolium chloride (TTC) staining at 21 days after MI revealed significant decrease in the scar sizes of infarcted hearts in AAV9-ALKBH5 mice compared to AAV9-control mice (Figure 5D-E). In addition, ALKBH5 overexpression significantly improved cardiac function and increased the numbers of pH3- and Aurora B kinase-positive cells (Figure 5F-I). In addition, we observed decreased expression of ALKBH5 in mouse hearts after MI (Figure S2A).

**Figure 6:**
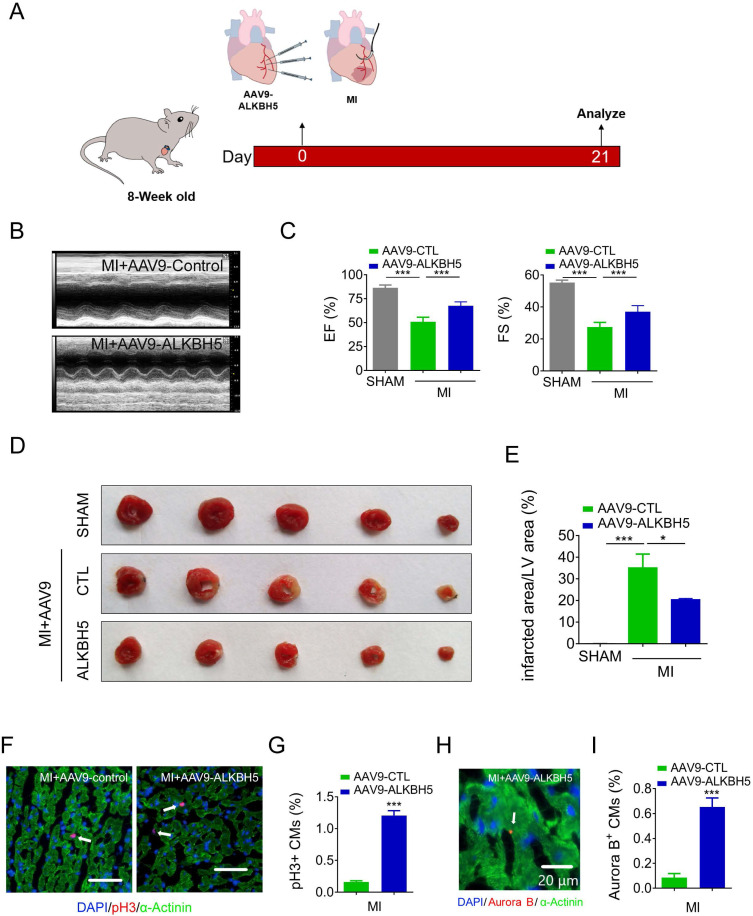
** Overexpression of ALKBH5 promotes heart regeneration of adult mice (8-week old) after MI.** (A) Schematic diagram for experimental procedure. (B, C) Cardiac function of AAV9-ALKBH5 mice and AAV9-control mice at day 21 after MI. n = 4 for SHAM mice, n = 6 for MI+AAV9-CTL mice and MI+AAV9-ALKBH5 mice. ***P < 0.001. (D, E) Infarct size of hearts in SHAM, MI+AAV9-ALKBH5 and MI+AAV9-control mice by 2,3,5-triphenyltetrazolium chloride (TTC) staining. **P < 0.05, ***P < 0.001. (F, G) pH3 immunofluorescence staining in MI+AAV9-ALKBH5 and MI+AAV9-control mice and quantification of pH3-positive CMs. (16725 CMs in the AAV9-control group and 17741 CMs in the AAV9-ALKBH5 group). ***P < 0.001. Scale bars, 50 µm. (H, I) Aurora B kinase immunofluorescence staining in hearts from MI+AAV9-ALKBH5 and MI+AAV9-control mice and quantification of pH3-positive CMs (7388 CMs in the AAV9-control group and 7107 CMs in the AAV9-ALKBH5 group). ***P < 0.001. Scale bar, 20 µm.

We then studied the effect of AAV9-ALKBH5 in adult (8-week old) mice hearts. The cardiac function did not change after transfection with AAV9-ALKBH5 for 21 days (Figure S3A-B). However, the CM surface size was decreased in the cardiac tissues after transfection with AAV9-ALKBH5 compared to those of AAV9-control mice (Figure S3C-D). PH3 staining also confirmed an increased in proliferative CMs in adult hearts after transfection with AAV9-ALKBH5 (Figure S3E-F). Next, we studied the role of ALKBH5 overexpression in adult mice after MI (Figure 6A). As expected, AAV9-ALKBH5 overexpressing mice exhibited significantly increased cardiac function at day 21 after MI (Figure 6B-C). Consistently, dramatically decreased myocardial scar sizes were observed in the hearts of AAV9-ALKBH5 overexpressing mice relative to those of AAV9-control mice, and ALKBH5 overexpression also increased the proliferative capacity of CMs (Figure 6D-I). These data indicate that ALKBH5 protects against MI injury by stimulating CMs to re-enter the cell cycle.

### ALKBH5 upregulates the translation but not the transcription of YAP

**Figure 7:**
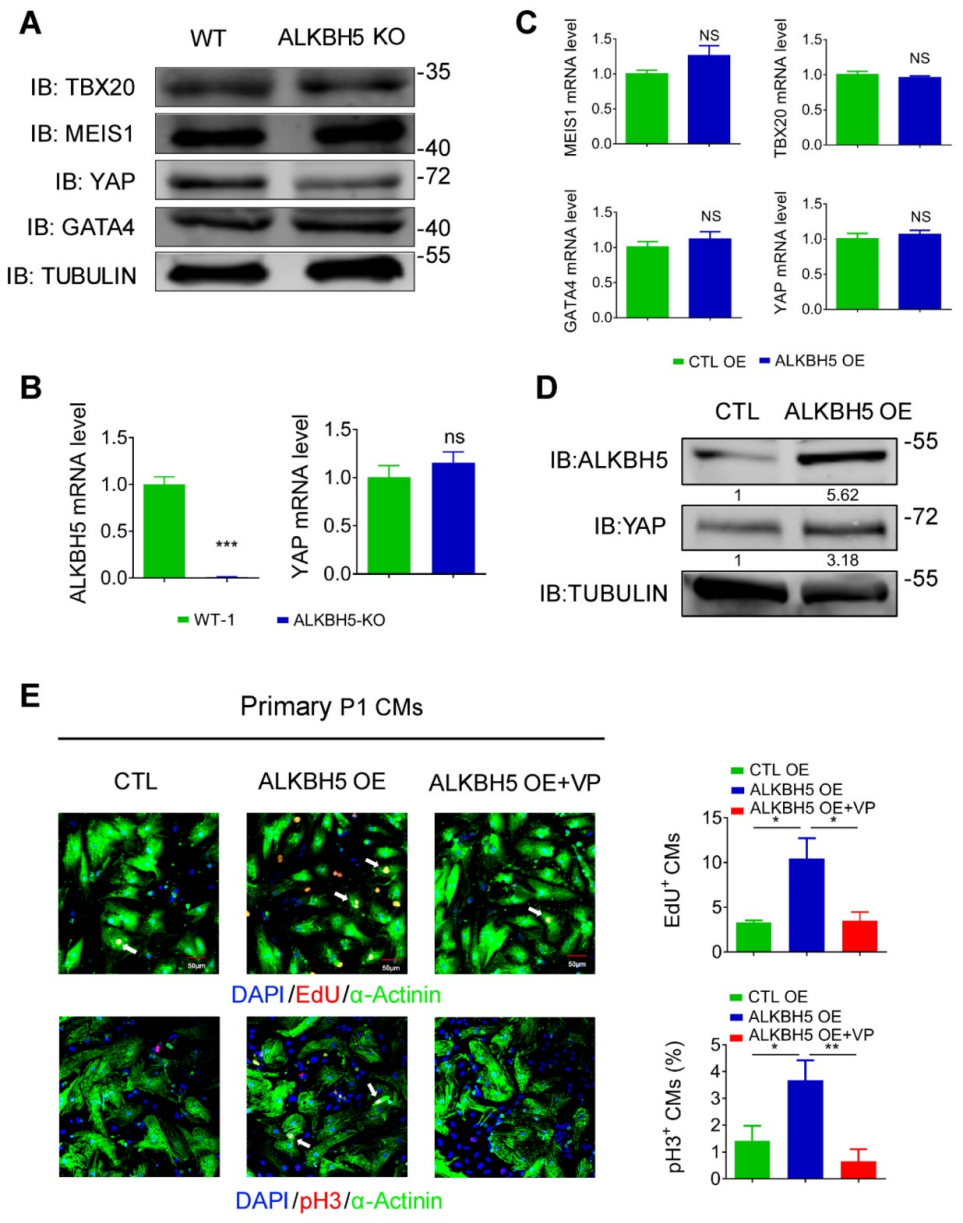
** ALKBH5 promotes translation of YAP.** (A) Western blot analysis of TBX20, MEIS1, YAP and GATA4 in hearts from WT and ALKBH5 KO mice (P1). (B) RT-qPCR analysis of ALKBH5 and YAP in hearts from WT and ALKBH5 KO mice. ***p < 0.001 (n = 4). (C) RT-qPCR analysis of TBX20, MEIS1, YAP and GATA4 in cultured P1 cardiomyocytes transfected with ALKBH5 OE plasmid (n = 4). (D) Western blot analysis of YAP and ALKBH5 in cultured P1 cardiomyocytes transfected with control or ALKBH5 OE plasmid. (E) Neonatal P1 cardiomyocytes in CTL, ALKBH5 OE and ALKBH5 OE+VP group were immunostained against EdU, phospho-histone H3 (pH3) and α-actinin (marks cardiomyocytes). DAPI was used for nuclear staining. n = 5 per group. *P < 0.05, **P < 0.01. The arrows point to EdU/pH3-positive signal. Scale bar, 50 μm.

To study how ALKBH5-mediated m^6^A modification regulates CM proliferation, we evaluated the protein expression of TBX20, MEIS1, YAP and GATA4, the core regulators of CM proliferation, in hearts from WT and *ALKBH5* KO mice. Notably, the level of YAP was dramatically reduced by *ALKBH5* KO, but that of TBX20, GATA4 and MEIS1 did not change (Figure 7A). Interestingly, knock out of ALKBH5 did not affect the transcription of the gene encoding YAP (Figure 7B). The mRNA of TBX20, MEIS1, YAP and GATA4 did not change in isolated neonatal CMs transfected with ALKBH5-expressing plasmids (Figure 7C). However, the protein level of YAP was significantly increased after overexpression of ALKBH5 (Figure 7D). Next, we further confirmed the regulation of YAP in ALKBH5-mediated cardiac regeneration in isolated neonatal CMs. The ALKBH5-induced CM proliferation could be reversed by the YAP-specific inhibitor verteporfin (VP; Figure 7E). These data suggested that ALKBH5-mediated m^6^A modification increases the translation of YAP without affecting its transcription, which determines cardiomyocyte proliferation.

### ALKBH5 upregulates the stability of YTHDF1 mRNA and promotes the efficient translation of YAP

**Figure 8:**
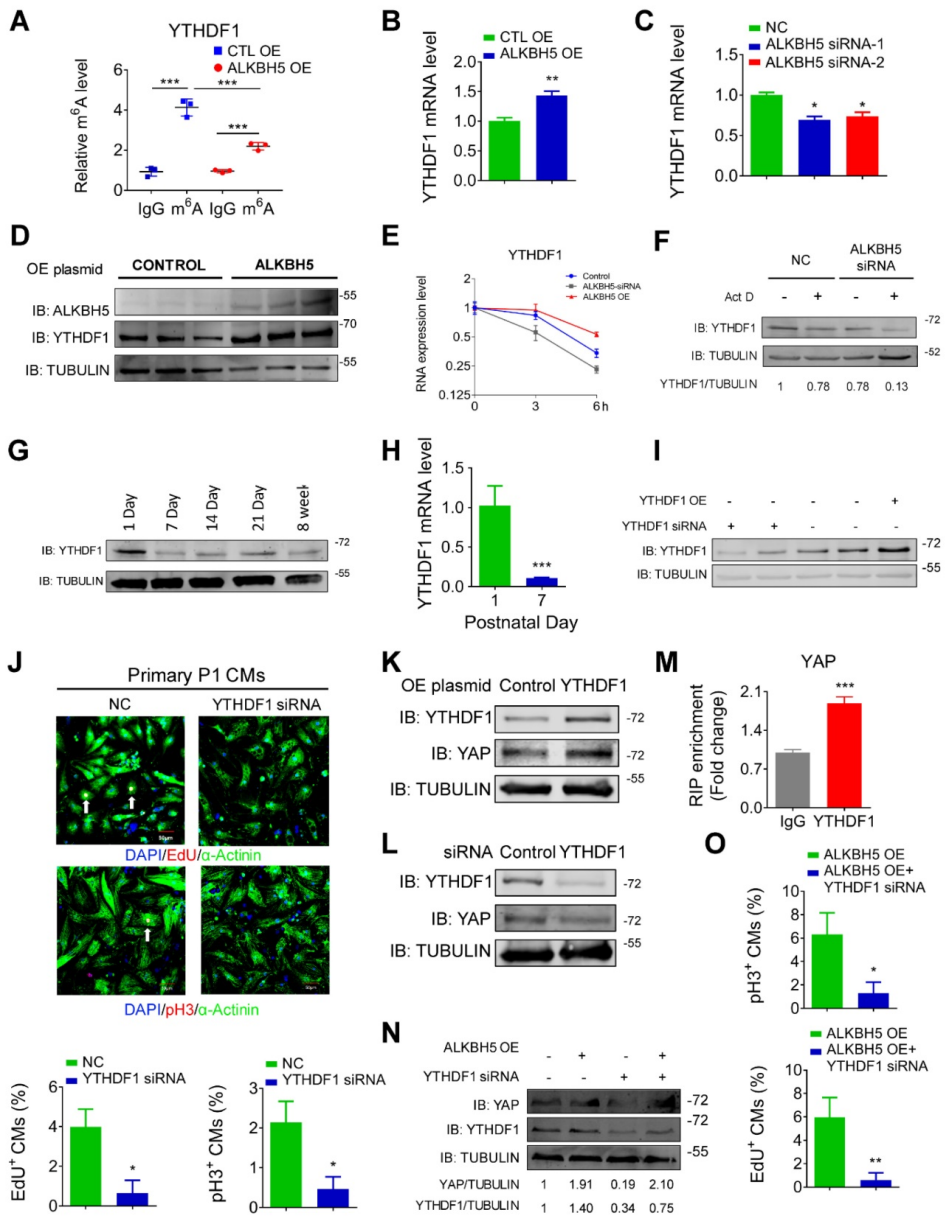
** ALKBH5 upregulates the mRNA stability of YTHDF1 in turn promotes the translation of YAP.** (A) m^6^A-meRIP-qPCR of YTHDF1 in CTL or ALKBH5 OE P1 CMs (***P < 0.001, n = 4). (B) RT-qPCR analysis of YTHDF1 in cultured P1 cardiomyocytes transfected with control or ALKBH5 OE plasmid (**P < 0.01, n = 4). (C) RT-qPCR analysis of YTHDF1 in cultured P1 cardiomyocytes transfected with control siRNA or ALKBH5 siRNAs (*P < 0.05, n = 4). (D) Western blot analysis of ALKBH5 and YTHDF1 in cultured P1 cardiomyocytes transfected with control or ALKBH5 OE plasmid. (E) RT-qPCR of YTHDF1 transcripts in Act D-treated control and ALKBH5 siRNA P1CMs (n = 4). (F) Western blot analysis of YTHDF1 in Act D-treated control or ALKBH5 siRNA P1 CMs (G) Western blot assay of YTHDF1 in hearts from P1, P7, P14, P21 and 8W mice. (H) RT-qPCR analysis of YTHDF1 in hearts from P1 and P7 cardiomyocytes (***P < 0.001, n = 4). (I) Western blot analysis of YTHDF1 in cultured P1 cardiomyocytes transfected with control, YTHDF1 siRNA or YTHDF1 OE plasmid. (J) Cardiomyocytes isolated from P1 mice were transfected with CTL-siRNA or YTHDF1-siRNA and immunostained against EdU, phospho-histone H3 (pH3) and α-actinin (marks cardiomyocytes). DAPI was used for nuclear staining. n = 5 per group. *P < 0.05. The arrows point to EdU/pH3-positive signal. Scale bar, 50 μm. (K) Western blot analysis of YAP in cultured P1 cardiomyocytes transfected with control or YTHDF1 OE plasmid. (L) Western blot analysis of YAP in cultured P1 cardiomyocytes transfected with control or YTHDF1 siRNA. (M) RIP-qPCR analysis of the interaction of YAP and YTHDF1 in hiPSC-CMs. Enrichment of YAP was normalized to input (***P < 0.001, n = 4). (N) Western blot analysis of YAP and YTHDF1 in CTL, ALKBH5 OE, YTHDF1 siRNA and ALKBH5 OE+YTHDF1 siRNA P1 CMs. (O) Neonatal P1 cardiomyocytes in ALKBH5 OE and ALKBH5 OE+YTHDF1 siRNA group were immunostained against EdU and pH3 (*P < 0.05, **P < 0.01). n = 5 per group.

To determine the upstream process by which ALKBH5 affects the efficiency of YAP translation, we subjected hiPS-CMs to a m^6^A transcriptomic microarray analysis to identify the downstream targets of ALKBH5. Notably, we observed fairly strong m^6^A methylation of YAP mRNA in the control group (~0.8% in mRNA), which was not affected by ALKBH5 overexpression (Figure S4A). Intriguingly, our microarray analysis revealed a significant decrease in the methylation of m^6^A reader YTHDF1, which is known to recognize and promote the translation of m^6^A methylated genes, in the ALKBH5 overexpression group (Figure S4A). This result was further supported by a m^6^A-specific qPCR analysis (Figure 8A). It is also showed that YTHDF1 gene carries several potential m6A modification sites according to a sequence-based m6A modification site predictor (http://www.cuilab.cn/sramp; Figure S4B). Moreover, ALKBH5 overexpression upregulated the expression of YTHDF1 mRNA, whereas ALKBH5 knockdown did the opposite (Figure 8B-C). ALKBH5 overexpression also led to an increase in the level of YTHDF1 protein (Figure 8D). As m^6^A modification modulates gene expression mainly by affecting the stability of mRNA, we measured the half-lives of YTHDF1 mRNAs in neonatal CMs after transfection with an ALKBH5-expressing plasmid or targeting siRNA. As expected, the half-life of YTHDF1 mRNA decreased remarkably in response to ALKBH5 knockdown (Figure 8E-F).

Next, we studied the role of YTHDF1 in CM proliferation. Our qPCR and Western blot analyses revealed fairly strong expression of YTHDF1 in P1 hearts, followed by a gradual decrease with increasing postnatal days (Figure 8G-H and Figure S4C). YTHDF1 knockdown noticeably inhibited the proliferation of isolated neonatal CMs (Figure 8I-J, and Figure S4D-E). We also found that knockdown of YTHDF2 could inhibit P1 cardiomyocyte proliferation (Figure S4E). In addition, forced YTHDF1 overexpression led to a significant increase in CM cell division (Figure S4F-H). The pH3 results showed that overexpression of YTHDF1 can rescue the proliferation in ALKBH5 KD CMs (Fig. S4I). Tunel assay showed that YTHDF1 KD did not affect the level of apoptotic cells (Figure S4J).

We also evaluated the potential influence of YTHDF1 on the YAP pathway. The level of YAP protein increased remarkably in response to YTHDF1 overexpression but decreased in response to YTHDF1 knockdown (Figure 8K-L). As expected, our RIP-qPCR analysis revealed an interaction of YAP with YTHDF1 in hiPS-CMs (Figure 8M). Moreover, YTHDF1 knockdown inhibited the translation of YAP and the proliferative capacities of neonatal CMs even in the presence of ALKBH5 overexpression (Figure 8N-O). The exogenous expression of either YAP or YTHDF1 induced a partial recovery of the proliferative capacities of CMs, regardless of the ALKBH5 level (Figure S5A-B). These results demonstrate that ALKBH5 increases the stability of YTHDF1 mRNA and consequently promotes the translation of YAP.

### The effects of ALKBH5 and YTHDF1 on human cardiomyocytes

**Figure 9:**
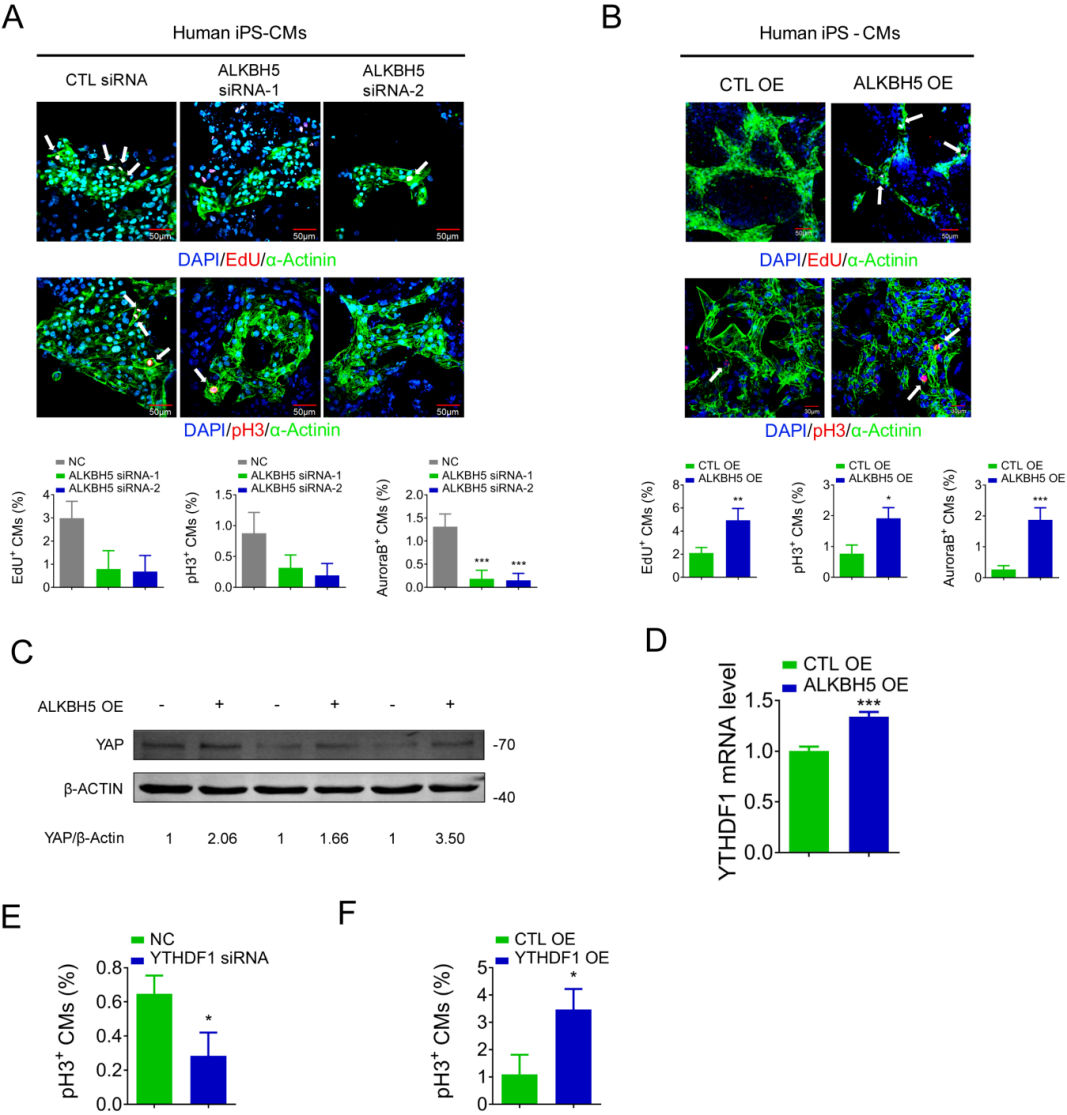
** ALKBH5 regulates proliferation of human cardiomyocytes.** (A) HiPSC-CMs were transfected with CTL-siRNA or ALKBH5-siRNAs for 48 hr. Cells were immunostained against EdU, phospho-histone H3 (pH3), Aurora B kinase and α-actinin (marks cardiomyocytes). DAPI was used for nuclear staining. n = 5 per group. ***P < 0.001. The arrows point to EdU/pH3/Aurora B kinase-positive signal. Scale bar, 50 μm. (B) HiPSC-CMs were transfected with CTL-plasmid or ALKBH5 OE plasmid for 48 hr. Cells were immunostained against EdU, phospho-histone H3 (pH3), Aurora B kinase and α-actinin (marks cardiomyocytes). DAPI was used for nuclear staining. n = 5 per group. *P < 0.05, **P < 0.01, ***P < 0.001. The arrows point to EdU/pH3/Aurora B kinase-positive signal. Scale bar, 50 μm. (C) Western blot analysis of YAP in hiPSC-CMs transfected with control or ALKBH5 OE plasmid. (D) RT-qPCR analysis of YTHDF1 hiPSC-CMs transfected with control plasmid or ALKBH5 expressing plasmid (***P < 0.001, n = 4). (E) HiPSC-CMs were transfected with CTL-siRNA or YTHDF1-siRNA for 48 hr. Cells were immunostained against phospho-histone H3 (pH3) and α-actinin (marks cardiomyocytes). DAPI was used for nuclear staining. n = 5 per group. *P < 0.05. (F) HiPSC-CMs were transfected with CTL-plasmid or YTHDF1 OE plasmid for 48 hr. Cells were immunostained against phospho-histone H3 (pH3) and α-actinin (marks cardiomyocytes). DAPI was used for nuclear staining. n = 5 per group. *P < 0.05.

To determine the relevance of ALKBH5 in the human heart, we evaluated the influence of ALKBH5 and YTHDF1 on the proliferation of hiPSC-CMs. The ALKBH5-expressing plasmids and siRNAs were transfected to hiPS-CMs, and the efficiency was approved (Figure S6A-B). ALKBH5 silencing inhibited the proliferation of human CMs, as indicated by decreases in the percentages of EdU-, pH3-, and Aurora B kinase-positive cells (Figure 9A). In contrast, overexpression of ALKBH5 promoted the proliferation of human CMs (Figure 9B). Moreover, overexpression of ALKBH5 led to significant increases in the expression of YAP protein and YTHDF1 mRNA (Figure 9C-D). We then thereafter assessed the effects of YTHDF1 on the proliferation of hiPS-CMs. Notably, pH3 staining revealed a marked decrease in hiPS-CM proliferation following YTHDF1 knockdown and an increase upon YTHDF1 overexpression (Figure 9E-F). These results indicate that the ALKBH5-YTHDF1-YAP axis enhances the proliferative capacity of CMs in both humans and mice.

## Discussion

The sustained CM losses and cardiac dysfunction associated with heart diseases are largely attributable to their extremely limited regenerative capacity 13-15. Therefore, strategies to promote the re-activation of adult CM proliferation after heart injury are particularly important. In this study, we found that the m^6^A eraser ALKBH5 played a vital role in CM proliferation and regeneration. Knockout of *ALKBH5* in mice markedly suppressed CM proliferation and regeneration, while AAV9-mediated overexpression of ALKBH5 increased the number of proliferating CMs, reduced scar sizes, and restored cardiac function after MI injury. Mechanistically, ALKBH5-mediated m^6^A demethylation upregulated the expression of YTHDF1, thus promoting the translation of YAP. These findings expand our understanding of the loss of regenerative capacity in the adult mammalian heart. Targeting ALKBH5 and YTHDF1 could pave the way for new treatments for MI and related ischemic injury.

Recently, m^6^A modification has been revealed to be critical in various biological processes. Dysregulated m^6^A modification has been linked to stem cell pluripotency maintenance and differentiation 16-19, DNA damage responses 20, memory formation 21, and disease progression in multiple cancers 22-24. In particular, three recent studies uncovered a regulatory role of m^6^A modification in the mammalian heart. Prabhu et al. reported that the expression of m^6^A eraser FTO was decreased in failing heart and hypoxic CMs. Enforced expression of FTO enhanced cardiac function after ischemia injury via improving the expression of cardiac contractile genes such as SERCA2a 25. Dorn et al. revealed important roles of METTL3-mediated m^6^A modification in cardiac hypertrophic stress responses. Enforced expression of METTL3 results in cardiac hypertrophy, whereas knockdown of METTL3 leads to maladaptive eccentric remodeling and heart failure 26. Moreover, Song et al. demonstrated the involvement of m^6^A in the regulation of ischemic heart disease by establishing a link between METTL3-ALKBH5 and autophagy^27^. Beyond these impressive findings, our data indicated that ALKBH5 has decreased expression in murine hearts after birth. Enforced expression of ALKBH5 promotes cardiomyocyte proliferation by enhancing the translation of YAP in an YTHDF1-dependent manner. Thus, the present study enhances our understanding of the relationship between m^6^A RNA modification and mammalian heart function.

The highly conserved Hippo signaling pathway is involved in the regulation of organ size, cell proliferation, and stem cell fate determination. Emerging studies have shown that the Hippo pathway plays a vital role in regulating CM proliferation and heart regeneration 5, 6. When the Hippo pathway is placed in the “off” condition, the downstream co-effector YAP is dephosphorylated and enters the nucleus thereby stimulating CM proliferation. In addition, the silencing of various upstream factors in the Hippo pathway, including Lats2, Mst1, or Salv, promotes the nuclear localization of YAP and increases the proliferative capacity of CMs 28, 29. Our study is the first to demonstrate the involvement of m^6^A methylation in YAP-mediated cardiac regeneration. In addition, this study is also the first to establish the interaction between m^6^A and YAP in the mammalian heart. Contrary to previous understanding, by using m^6^A microarray and merip-qPCR assay, we demonstrated that the ALKBH5 expression status did not affect the m^6^A methylation level of our target gene YAP. Instead, ALKBH5-mediated m^6^A demethylation indirectly regulates the efficiency of YAP translation by modulating the expression of YTHDF1, thereby enhancing the stability of YTHDF1 mRNA.

The specific m^6^A binding protein YTH domain family protein 2 (YTHDF2) is known to localize m^6^A-modified mRNAs into decay sites and affect their stability. Unlike YTHDF2, YTHDF1 has a well-defined role in recognizing the m^6^A modification and promoting the translation of target transcripts by recruiting translation initiation factors 30. Recent studies have linked the dysregulation of YTHDF1 to the nervous system, cancer, phase separation, the immune system, and many other biological processes 31-33. However, whether YTHDF1 is involved in the regulation of the mammalian heart remains largely unclear. It is interesting to note that our data revealed the enrichment of YTHDF1 in neonatal heart, which was decreased after birth. Besides, YTHDF1 has the ability to promote cell division in both cultured murine CMs and human induced pluripotent stem cell derived CMs. Previous studies have demonstrated that abnormal m^6^A modification modulates the expression of target genes. Our results reveal the regulatory relationship between m^6^A eraser ALKBH5 and reader YTHDF1, which may help deepen our understanding of the molecular mechanism of m^6^A methylation.

This study had two major limitations. First, we focused on the role of ALKBH5-mediated m^6^A modification in CM proliferation, but did not conduct experiments to evaluate the effects of other m^6^A writers (e.g., METTL3, METTL14, WTAP) or erasers (e.g., FTO) on the regenerative capacity of CMs. Second, we only observed the short-term (<1 month) effects of ALKBH5 on CM proliferation. Future studies should verify the long-term effects of ALKBH5 on cardiac regeneration.

In summary, our study demonstrated a role for ALKBH5 in CM proliferation and highlighted the importance of m^6^A modification in cardiac regeneration. These findings expanded our understanding of the loss of regenerative capacity in the adult mammalian heart and may contribute to a strategy for restoring injured cardiac tissue.

## Materials and Methods

### Animal studies

Neonatal (within 3 days after birth) or adult (8-week old) C57BL/6 mice were obtained from the Animal Center at the Second Affiliated Hospital of Harbin Medical University. The animals in different experimental groups had similar body weights and physiological conditions.

The animal experiments were approved by the Ethics Committee of Harbin Medical University and were performed in accordance with the Guide for the Care and Use of Laboratory Animals, published by the US National Institutes of Health (Bethesda, MD, USA).

### Generation of *ALKBH5* knockout mice

*ALKBH5* knockout mice were generated on a C57BL/6J background GemPharmatech Co. Ltd (Jiang Su, China). In brief, a single guide RNA (sgRNA) targeting exon 1 of the *ALKBH5* transcript (ENSMUST00000044250.3) was transcribed *in vitro*. Next, Cas9 and sgRNA were microinjected into the fertilized eggs of C57BL/6J mice, which were then transplanted to obtain positive F0 mice. The statuses of F0 mice were confirmed by PCR and sequencing. Next, positive F0 mice were mated with C57BL/6J mice to yield stable F1 generation mice. F1 and F2 transgenic mice were used in this study. The following primers were used to detect wild-type (WT) mice: forward, 5´-GACAGCAAGGATATGGGCCAAT-3´ and reverse, 5´-CCCATATTAGGCTGGCACTTCT-3´. The following primers were used to detect KO mice: forward, 5´-TGGATTACCACCAACACGAATGG-3´ and reverse, 5´-GCTCCAGCTTCACGAGTTTGAG-3´.

### Primary cardiomyocyte culture

Primary CMs were dissected from the hearts of neonatal P1 or P7 mice. The cells were then subjected to several rounds of dissociation and digestion using a trypsin-EDTA solution (Solarbio, CHINA). Next, we collected the supernatants acquired after each round of digestion in Dulbecco's modified Eagle's medium (DMEM) supplemented with 10% fetal bovine serum (FBS) and 1% penicillin-streptomycin liquid. The total cell suspensions were centrifuged at 1700 rpm for 7 min. After removing the supernatant, the cells were re-suspended in DMEM medium supplemented with 10% FBS and 1% penicillin-streptomycin liquid for 90 min. During this process, the majority of non-CMs adhered to the culture bottle, while the CMs remained in suspension. The suspended cells were collected and incubated on culture plates at certain densities.

### Verteporfin treatment

Primary CMs were isolated from the hearts of neonatal P1 mice as described above. Verteporfin (Sigma-Aldrich, USA) was dropped into cell culture medium at a concentration of 1 μM.

### TUNEL Assay

We performed TUNEL assay to detect cell apoptosis by using an *in situ* cell death detection kit (Roche, Germany). In brief, cells were fixed with 4% paraformaldehyde (PFA) for 15min. The cells were then incubated with blocking buffer before permeabilized with 0.1% Triton X-100. The cells were then treated with TUNEL reaction mixture for 1 h. The nuclei were stained with DAPI.

### HiPSC cardiomyocyte differentiation

HiPSCs were maintained in E8 medium as described previously 34. Small molecule compounds were added to the cultures to induce differentiation into CMs. When the cells reached ~60% confluence, we directed differentiation in RPMI 1640+B27-insulin and supplemented the cells with 6 μM CHIR99021 on day 1. On days 2 and 3, the medium was changed to RPMI 1640+B27-insulin without CHIR99021. Subsequently, the medium was changed to RPMI 1640+B27 without insulin and supplemented with Wnt-C59 on days 3-5. On days 5-7, the medium was replaced with RPMI 1640 and B27 without insulin. From day 7, the cells were cultured with B27 until they began to contract.

### Apical resection

Neonatal P1 mice were anesthetized on an ice bed until they were sluggish. Following a skin incision, the intercostal muscles were subjected to blunt dissection to expose the heart at the fourth intercostal space. Iridectomy scissors were used to resect the apices of P1 mice hearts quickly and minimize the duration of surgery in an attempt to improve the survival rate. After apical resection, the skin wounds were closed using skin adhesive, and the neonates were placed in a warm environment (37 ºC) for several minutes until recovery. Sham-operated mice were exposed to the same procedure without apical resection. Hearts were collected 21 days after apical resection.

### Myocardial infarction

Juvenile mice (P7) were anesthetized on an ice bed. Adult mice (8-week old) required tracheal intubation and were intraperitoneally anesthetized using 2,2,2-tribromoethanol (i.e., Avertin). Next, the left coronary artery was ligated with a 6-0 prolene suture, and 4.26 × 10^10^ viral genome particles of AAV9-ALKBH5 or AAV9-control in a total volume of 30 μL were injected into the myocardium bordering the infarct zone of each adult mouse at three sites after myocardial infarction (MI). P7 mice were injected at two sites with 2.84 × 10^10^ AAV9-ALKBH5 or AAV9-control viral genome particles in a volume of 10 μL. Sham-operated mice underwent analogous surgical operations without occlusion of the coronary artery. After chest closure, the mice were then warmed until recovery.

### Immunohistochemistry

Fresh cardiac tissues were collected and embedded in optimal cutting temperature medium, and frozen at -80 ºC. The frozen tissues were then sectioned at a thickness of 8-µm using a cryostat. The tissue samples were fixed in chilled acetone for 10 min and then soaked with 3% H_2_O_2_ at 4 ºC for 10 min and permeabilized with 0.5% Triton X-100 for 60 min at 4 ºC. Subsequently, the samples were incubated in ready-to-use goat serum for 10 min at room temperature and subsequently incubated overnight at 4 ºC with primary antibodies diluted in 0.8% Triton X-100. Subsequently, the samples were washed in phosphate-buffered saline (PBS) and stained with secondary antibodies for 1 h at room temperature in the dark. Finally, the cells were stained with 4′,6-diamidino-2-phenylindole dihydrochloride (DAPI) for 15 min in the dark to visualize the nuclei. Images were captured using a fluorescent microscope (Olympus, JAPAN). We used ImageJ software to count the number and size of the cardiomyocytes. The antibodies are listed in Table S2.

### Histology

Heart tissues were fixed with 4% paraformaldehyde (PFA) for 24 h. Following dehydration and paraffin embedding, the tissues were cut into serial 6-μm sections from the cardiac apex to base at 0.4-mm intervals. Next, hematoxylin and eosin (HE) and TTC staining were used to evaluate the degree of MI in each heart. All staining steps were performed according to our previous study 12. The scar sizes in the various sections were measured using Image Pro Plus software.

### Echocardiography

The mice were anesthetized intraperitoneally with Avertin. Cardiac function was measured via transthoracic echocardiography using a Vevo 1100 Visual Sonics device equipped with a 30-MHz transducer (RMV-707B, Visual Sonics, Toronto, ON, Canada).

### m^6^A-mRNA&lncRNA Epitranscriptomic microarray

Total RNAs were isolated after transfection with ALKBH5 expressing plasmids and the quality of total RNAs were analyzed using a NanoDrop ND-1000. The extracted RNAs were processed with anti-m^6^A antibody in order to obtain the m6A modified RNAs. Finally, we tagged the "IP" (the m^6^A modified RNAs from mixing with the magnetic beads)” RNAs and “the Sup” (unmodified RNAs from the supernatant) RNAs with Cy5 and Cy3, respectively, as cRNAs using an Arraystar Super RNA Labeling Kit. Next, hybridization of cRNA to Arraystar Human mRNA&lncRNA Epitranscriptomic Microarray (8x60K, Arraystar) was performed for sequencing and the result was detected using an Agilent Scanner G2505C and analyzed using the Agilent Feature Extraction software (version 11.0.1.1). The raw intensities of “IP” and “Sup” were normalized with the average of the log2-scaled Spike-in RNA intensities. The m^6^A methylation level was built with the IP (Cy5-labeled) and Sup (Cy3-labeled) normalized intensities; m^6^A methylation was built with the IP (Cy5-labeled) normalized intensities. The distinguishable m^6^A-methylation pattern was then depicted by hierarchical clustering.

### Western blotting

Proteins were extracted from cells or tissue samples as described previously^34^. The proteins were quantified using a BCA protein assay kit (Beyotime) and resolved using sodium dodecyl sulfate-polyacrylamide gel electrophoresis (SDS-PAGE) with 10%-12.5% gels. The separated proteins were transferred to a methanol-activated nitrocellulose (NC) filter membrane (PALL). Major antibodies specific for the following proteins were used for immunoblotting: YAP (1:1000 dilution; Santa), ALKBH5 (1:1000; Millipore), and tubulin (1:5000; Affbiotech).

### Quantitative RT-PCR

Total RNA was isolated from cells and tissue samples using TRIzol reagent (Ambion/Life Technologies, Carlsbad, CA, USA). Total RNA was dissolved in RNase-free water (DEPC) and reverse-transcribed to cDNA. Quantitative RT-PCR was then performed using FastStart Universal SYBR Green Master (Rox). Each sample was subjected to quadruplicate analyses. The primers are listed in Table S1.

### RIP-qPCR

The RNA immunoprecipitation experiment was performed using antibodies against m^6^A or YTHDF1 or a negative IgG control and a Magna RIP kit (Millipore, cat. 17-700). In brief, the cells were lysed in RIP lysis buffer, then the mixed antibodies and samples were immunoprecipitated using A/G magnetic beads, and a magnetic frame was used to fix the magnetic bead-bound compounds and to enable the unbound substance to be washed away. RNA was extracted and purified according to the manufacturer's instructions. Finally, the purified RNA was subjected to a RT-qPCR analysis. The primers are listed in Table S1.

### m^6^A dot blot assay

Poly-A+ mRNA were isolated using a PolyATtract® mRNA Isolation Systems (Promega) in accordance with the instructions. First, the mRNA sample was applied to a N+ nylon membrane (RPN303B, BIOSHARP, China) and UV-crosslinked using a crosslinking device (CL-1000, UVP). Next, the membranes were incubated overnight using an anti-m^6^A antibody (1:500 dilution in PBS; Synaptic Systems, catalog number: 202003) after blocking in 5% milk for 1 h at room temperature. The membranes were then incubated with secondary antibodies and exposed using an Odyssey device (LI-COR Biosciences, Lincoln, NE, USA).

### m^6^A quantification (m^6^A-ELISA assay)

The quantification of the m^6^A RNA methylation levels in differentiating hESCs was detected using an m^6^A RNA Methylation Quantification Kit (Abcam, ab185912) as described by the manufacturer. In brief, messenger RNA was isolated from cells and bound to a strip well for 90min. Each well was washed and the captured antibody, detection antibody, and enhancer antibody were added. Color developing solution was then added and the absorbance at 450 nm was measured. Finally, to determine the relative m^6^A RNA methylation status, the calculation for the percentage of m^6^A in RNA was carried out using the following formula:


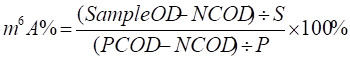


Where S is the amount of input sample RNA in ng and P is the amount of input positive control in ng.

### Cell transfection

Lipofectamine RNAiMAX (1378-150, Invitrogen) was used to deliver small interfering RNAs (siRNAs) into neonatal CMs while ViaFect Transfection Reagent (E4982, Promega) was used to transfect neonatal CMs with overexpression plasmids. The transfection methods were carried out according to the manufacturer's instructions. The transfected cells were analyzed after 48 h. The sequences of siRNAs are listed in Table S3.

### Immunofluorescence assay

Three steps were required before immunostaining: fixation, penetration and blocking at 15, 45, and 30 min respectively. The fixative fluid was 4% paraformaldehyde, the penetrating fluid was PBS containing 0.3% Triton-X100, and the blocking solution was goat serum. The sample was stained with primary antibody overnight at 4 ºC. Next, the sample was incubated with the secondary antibody and DAPI on the following day. Images were captured using confocal microscopy (FV1000, Olympus). The antibodies are listed in Table S2.

### Statistics

Data were presented as mean ± SD. Comparison between each group was performed using t-test, and one-way or two-way analysis of variance followed by Tukey's post-hoc analysis. Statistical analysis was performed using Prism 7.0 software. *P < 0.05, **P < 0.01, ***P < 0.001. Differences were considered significant when P < 0.05.

## Supplementary Material

Supplementary figures and tables.

## Abbreviations

AR: apex resection
DMEM: Dulbecco's modified Eagle's medium
HE: hematoxylin and eosin
MI: myocardial infarction
WT: wild-type
YAP: yes-associated protein
