# Supplementary material for: ALKBH5 regulates cardiomyocyte proliferation and heart regeneration by demethylating the mRNA of YTHDF1
Source: Theranostics. 2021 Jan 1;11(6):3000–16. doi: 10.7150/thno.47354 (PMC7806463; doi:10.7150/thno.47354)
Supplement: Supplementary file 1 — Supplementary figures and tables. [file thnov11p3000s1.pdf]

Supplementary Materials

Figure S1

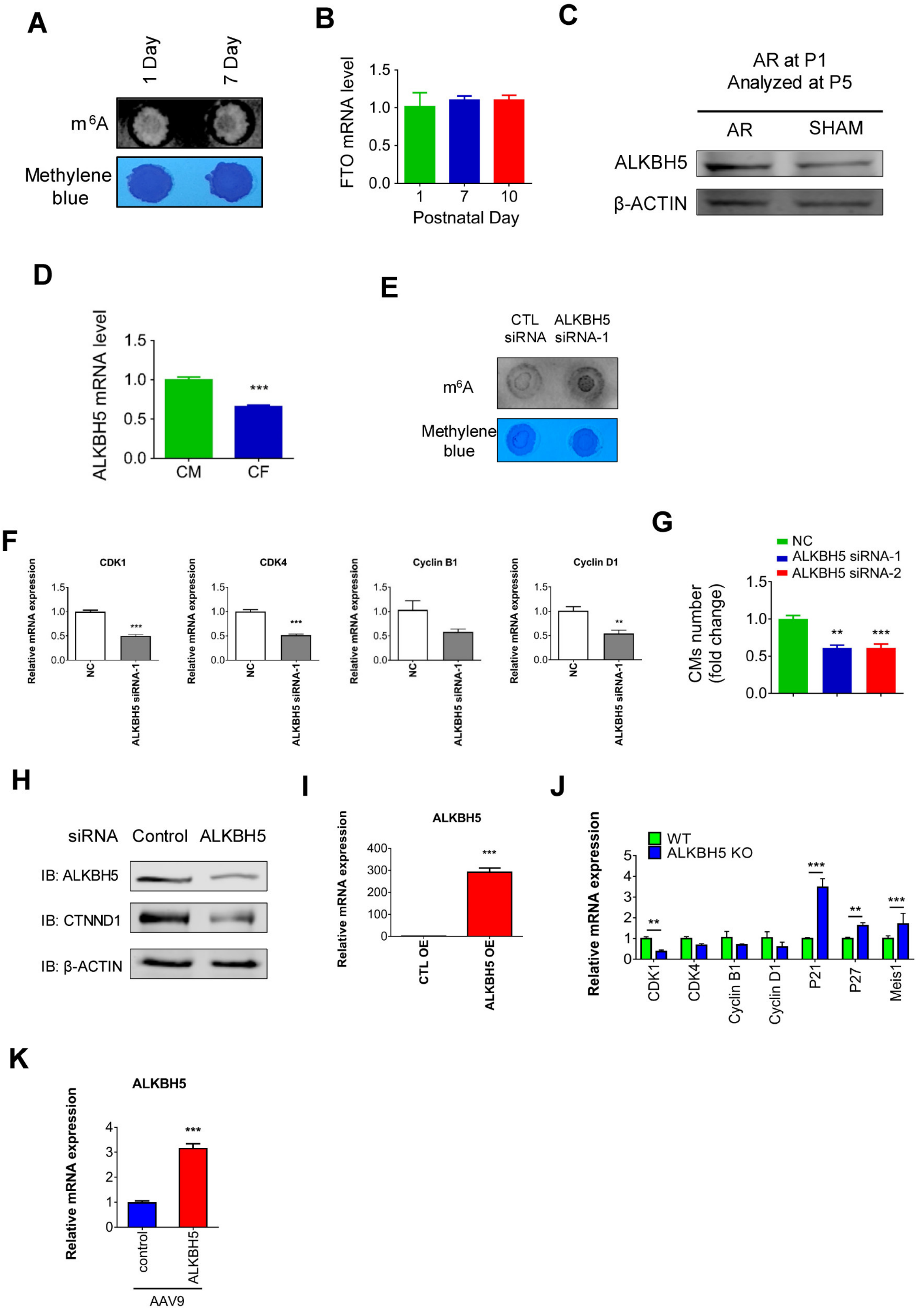

Figure S1. ALKBH5 regulates cardiomyocytes proliferation. (A) m^6A dot blot assay of mRNAs isolated from P1 and P7 hearts. (B) RT-qPCR analysis of FTO in hearts from P1, P7 and P10 mice (n = 4). (C) Western blot analysis of ALKBH5 of hearts harvested 4 days after an AR operation on a 1-day-old mice. (D) RT-qPCR analysis of ALKBH5 expression in cultured P1 cardiomyocytes (CM) and cardiac fibroblasts (CF). (***p < 0.001, n = 4). (E) m^6A dot blot assay of control and ALKBH5 siRNA-1 cells. (F) RT-qPCR analysis of CDK1, CDK4, Cyclin B1 and Cyclin D1 in cultured P1 cardiomyocytes transfected with control siRNA or ALKBH5 siRNA (**p < 0.01, ***p < 0.001, n = 4). (G) Quantification of the number of myocytes per microscopic field from control or ALKBH5-siRNA treated cells (n = 4). (H) Western blot analysis of ALKBH5 and CTNND1 in cultured P1 cardiomyocytes transfected with control or ALKBH5 siRNA. (I) RT-qPCR analysis of ALKBH5 in cultured P1 cardiomyocytes transfected with control ALKBH5 expressing plasmid (***p < 0.001, n = 4). (J) RT-qPCR analysis of cell proliferation related genes in hearts from WT or ALKBH5 KO mice (**p < 0.01, ***p < 0.001, n = 4). (K) RT-qPCR analysis of ALKBH5 in hearts from P28 mice after injected with AAV9-ALKBH5 or AAV9-control at P7 (***p < 0.001, n = 4).

Figure S2

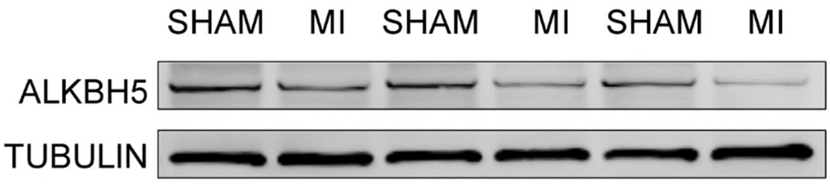

Figure S2. Decreased expression of ALKBH5 after MI. Western blot analysis of ALKBH5 in hearts from mice at 5 days after MI.

Figure S3

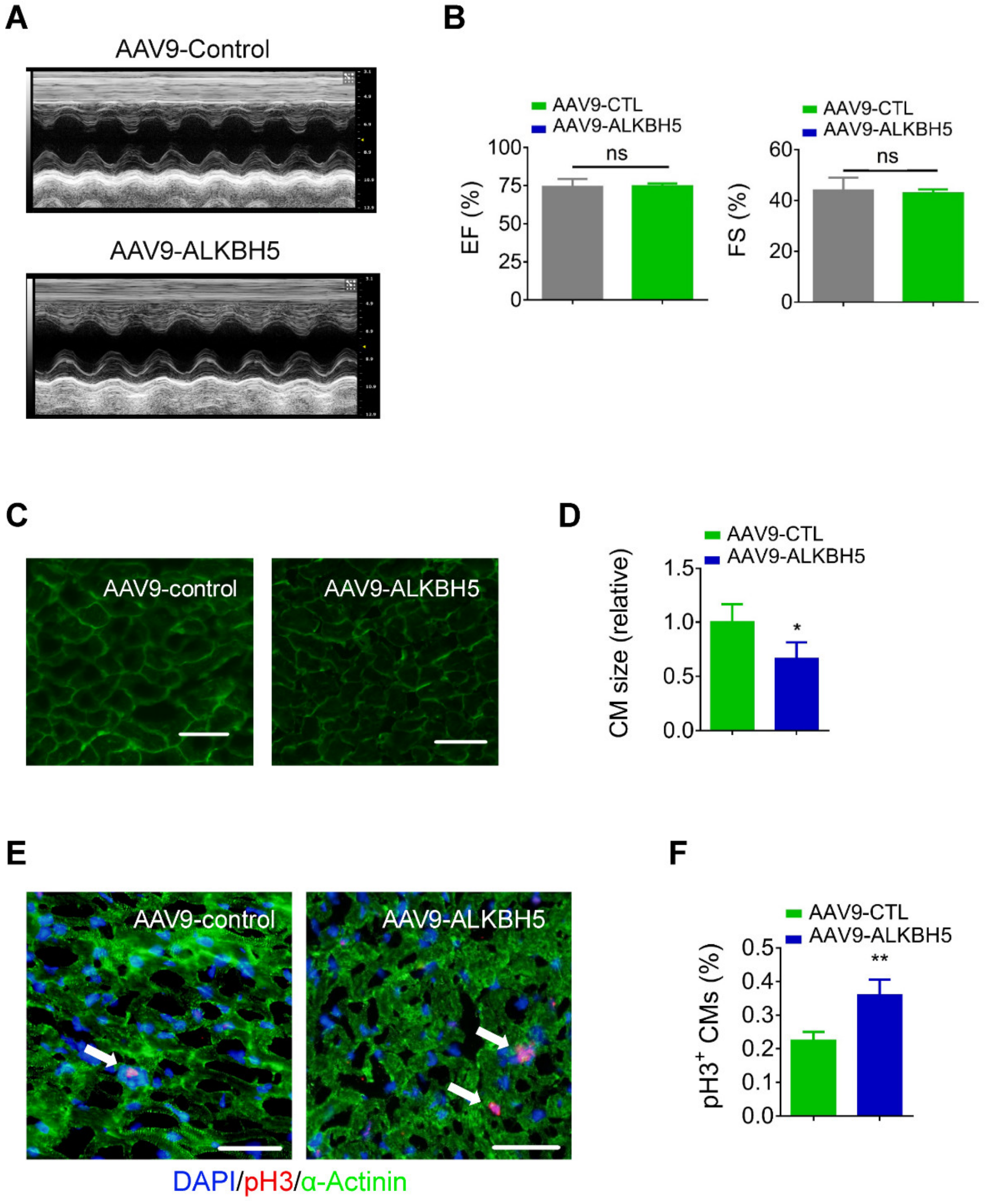

A
AAV9-Control
AAV9-ALKBH5
B
AAV9-CTL
AAV9-ALKBH5
ns
EF (%)
100
75
50
25
0
FS (%)
60
40
20
0
C
AAV9-control
AAV9-ALKBH5
D
CM size (relative)
1.5
1.0
0.5
0.0
*
E
DAPI/pH3/α-Actinin
F
pH3+ CMs (%)
0.5
0.4
0.3
0.2
0.1
0.0
**

Figure S3. ALKBH5 promotes cardiomyocyte proliferation in adult (8-weeks old) mice. (A, B) Cardiac function of adult mice after transfection with AAV9-CTL or AAV9-ALKBH5 for 21 days. n = 6. (C, D) Wheat germ agglutinin (WGA) staining and quantification of adult AAV9-ALKBH5 and AAV9-control mice hearts (*P < 0.05, n = 6). (E, F) PH3 immunofluorescence staining in adult AAV9-ALKBH5 and AAV9-control hearts and quantification of pH3-positive CMs (7953 CMs in the AAV9-control group and 10078 CMs in the AAV9-ALKBH5 group). **P < 0.01.

Figure S4

A

GeneSymbol	OE(Methylation_level)	CTL(Methylation_level)	description
YAP1	0.859338627	0.881984701	Yes associated protein 1 [Source:HGNC Symbol;Acc:HGNC:16262]
YTHDF1	0.291457145	0.580011436	YTH N6-methyladenosine RNA binding protein 1 [Source:HGNC Symbol;Acc:HGNC:15867]

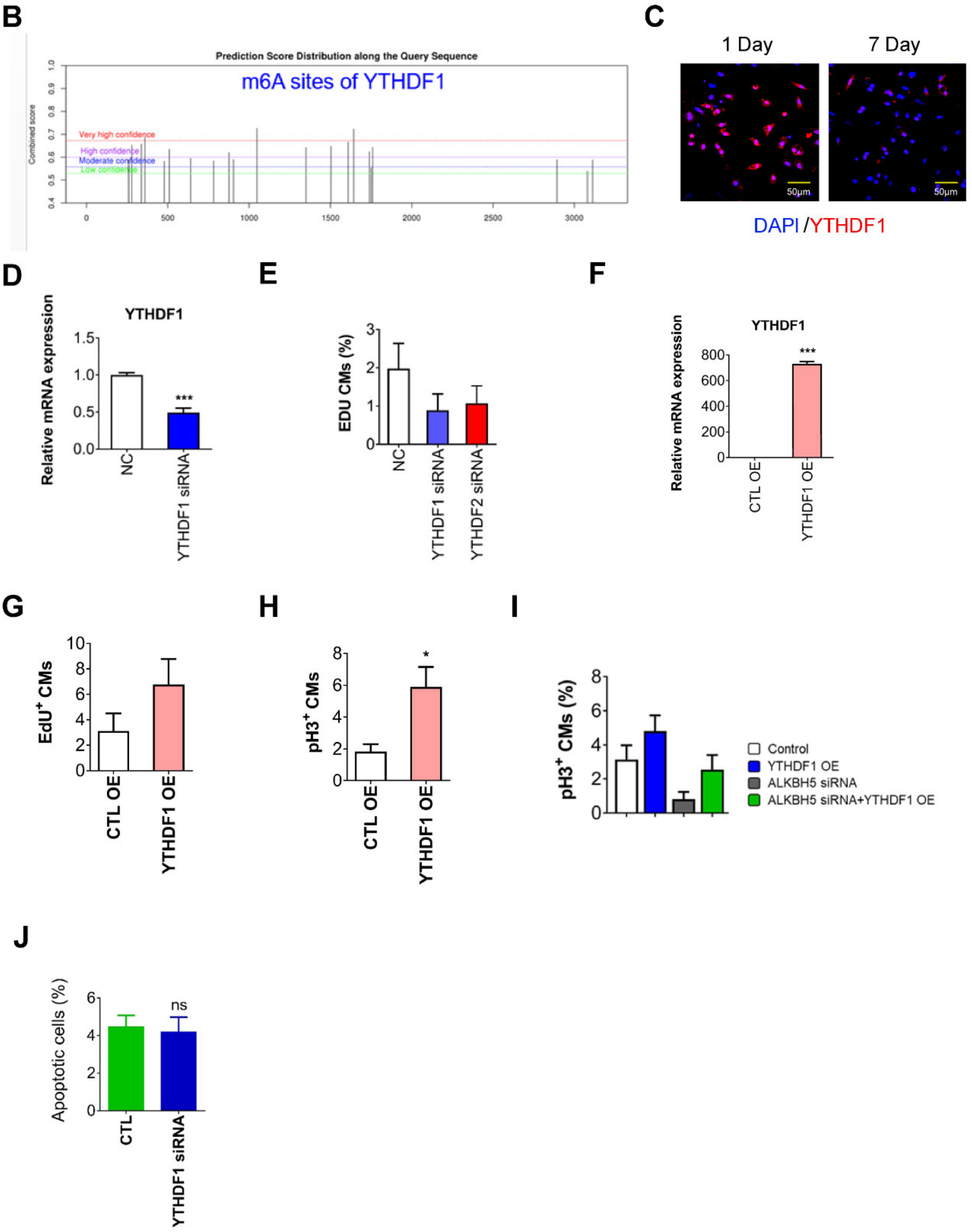

Figure S4. YTHDF1 promotes cardiomyocyte proliferation. (A) The methylation level for YAP and YTHDF1 in m^6A transcriptomic array. (B) Potential sites for m^6A modification in the sequence of YTHDF1 gene. (C) Immunostaining of YTHDF1 in cardiomyocytes isolated from P1 and P7 mice. Scale bar, 50 μm. (D) RT-qPCR analysis of YTHDF1 in cultured P1 cardiomyocytes transfected with control or YTHDF1 siRNA. ***$p < 0.001$, $n = 4$. (E) Cardiomyocytes isolated from P1 mice were transfected with YTHDF1 or YTHDF2 siRNAs and immunostained against EdU. (F) RT-qPCR analysis of YTHDF1 in cultured P1 cardiomyocytes transfected with control or YTHDF1 expressing plasmid. ***$p < 0.001$, $n = 4$. (G, H) Cardiomyocytes isolated from P1 mice were transfected with control plasmid or YTHDF1 expressing plasmid and immunostained against EdU or pH3. *$P < 0.05$. (I) Neonatal P1 cardiomyocytes in control, YTHDF1 OE, ALKBH5 siRNA and ALKBH5 siRNA+YTHDF1 OE group were immunostained against pH3. (J) The effect of YTHDF1 KD on apoptosis of P1 cardiomyocytes as determined by TUNEL staining.

Figure S5

A

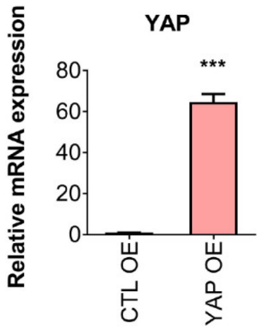
YAP
***
Relative mRNA expression
80
60
40
20
0
CTL OE
YAP OE

B

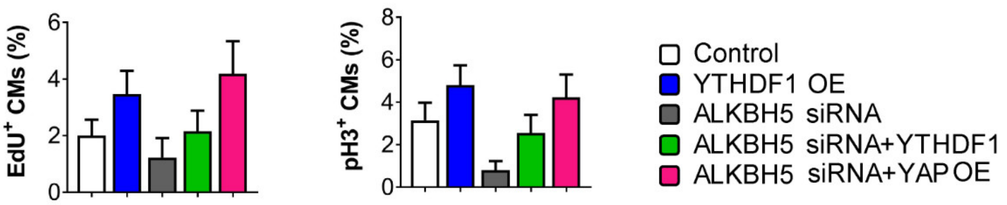
EdU+ CMs (%)
6
4
2
0
pH3+ CMs (%)
8
6
4
2
0
Control
YTHDF1 OE
ALKBH5 siRNA
ALKBH5 siRNA+YTHDF1
ALKBH5 siRNA+YAP OE

Figure S5. (A) RT-qPCR analysis of YAP in cultured P1 cardiomyocytes transfected with control or YAP expressing plasmid. (B) Cardiomyocytes isolated from P1 mice were transfected with YTHDF1 OE, ALKBH5 siRNA, ALKBH5 siRNA+YTHDF1 OE, ALKBH5 siRNA+YAP OE and immunostained against EdU or pH3.

Figure S6

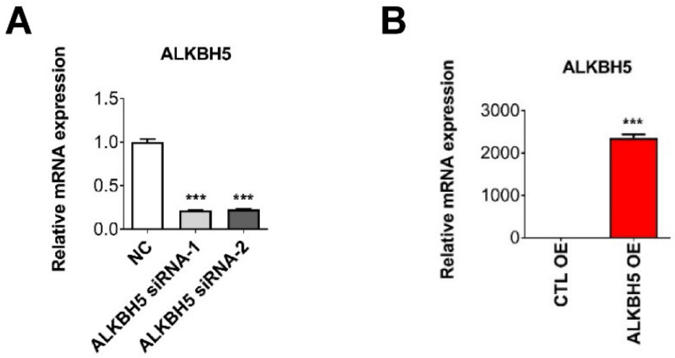

Figure S6. (A) RT-qPCR analysis of ALKBH5 in hiPSC-CMs transfected with control siRNA or ALKBH5 siRNAs (***p < 0.001, n = 4). (B) RT-qPCR analysis of ALKBH5 mRNA expression in hiPSC-CMs transfected with control plasmid or ALKBH5 plasmid (***p < 0.001, n = 4).

Supplementary Tables

Table S1. Primers used in RT-qPCR.

**Gene**	**Forward 5'-3'**	**Reverse 5'-3'**
**RT-qPCR**		
mALKBH5	GTGGGACCTTTTGGGTTTCAG	GCATACGGCCTCAGGACATTA
mYAP	ACCCTCGTTTTGCCATGAAC	TGTGCTGGGATTGATATTCCGTA
mCDK1	GTCCGTCGTAACCTGTTGAG	TGACTATATTTGGATGTCGAAG
mCDK4	AGTCAGTGGTGCCAGAGAT	AGATTCGCTTATGTGGGTTA
mCyclin B1	TATGAGCCTCGAATCCACATAGT	CCTCGTTCTGATAAGCAGTCAC
mCyclin D1	AGCCAGCTGCAGTGCTGTAC	CTGGTGGTGCCCGTTTTG;
mYTHDF1	TGGGAGTGGACATTTCTGTG	TTCTAAGGGCACCTCCTGTG
mYTHDF2	CAGGCATCAGTAGGGCAACA	TTATGACCGAACCCACTGCC
18s	CCTGGATACCGCAGCTAGGA	GCGGCGCAATACGAATGCCC
hALKBH5	GGCCGTATGCAGTGAGTGATT	TGTCCGTGTCCTTCTTTAGCG
hYTHDF1	ACCTGTCCAGCTATTACCCG	TGGTGAGGTATGGAATCGGAG
hYAP	TGCGTAGCCAGTTACCA	GGTGCCACTGTTAAGGA

Table S2. Antibodies and other reagents used in this study.

REAGENT or RESOURCE	SOURCE	IDENTIFIER
Primary antibodies		
Anti-phospho Histone H3	Millipore	06-570
Anti-Aurora B	Abcam	Ab2254
Anti-Tubulin alpha Ab	Affinity	AF7010
Anti-YAP	Santa Cruz	sc-101199
Anti-YTHDF1	Proteintech	17479-1-AP
Anti-m^6A	Synaptic System	No.202 003
Anti-Sarcomeric Alpha Actinin	Abcam	ab9465
Anti-β-actin	ZHONG SHAN	TA-09
Anti-ALKBH5	Millipore	ABE547
normal mouse IgG	Millipore	AP124P
normal rabbit IgG	Millipore	AP132P
Secondary antibodies		
IRDye® 800CW Goat anti-Mouse	LI-COR	925-32210
IRDye® 800CW Goat anti-Rabbit	LI-COR	925-32211
Alexa Fluor 488 Goat Anti-Mouse	Abcam	ab150113
Alexa Fluor 594 Goat Anti-Rabbit	Abcam	Ab150080
Chemicals and Peptides		
Essential 8 medium	STEMCELL Technology	05990
Accutase	Sigma-Aldrich	A6964
Matrigel matrix	Corning	354277
ROCK inhibitor	MedChemExpress	HY-10319
EDTA	Cellapy	CA3001500
CHIR-99021	MedChemExpress	HY-10182
B-27™ Supplement (50X)	Thermo Fisher	17504044
B-27™ Supplement, minus insulin	Thermo Fisher	A1895601
Wnt-C59	MedChemExpress	HY-15659
RPMI 1640 Medium	Thermo Fisher	22400105
Verteporfin	Simga	SML0534
Lipofectamine® RNAiMAX	Invitrogen	13778-150
ViaFect™ Transfection Reagent	Promega	E4982
Actinomycin D	MCE	HY-17559
Foetal Bovine Serum	BI	04-001-1ACS
DMEM	CORNING	10-013CVRC

Table S3. Target sequences of siRNAs used for specific gene knockdown experiments.

	siRNA Oligonucleotides	
Negative control-siRN	Sense	5'-UUCUCCGAACGUGUCACGUTT-3'
	Antisense	5'-ACGUGACACGUUCGGAGAATT-3'
hALKBH5 siRNA-1	Sense	5'-CUGCGCAACAAGUACUUCUTT-3'
	Antisense	5'-AGAAGUACUUGUUGCGCAGTT-3'
hALKBH5 siRNA-2	Sense	5'-GGAUAUGCUGCUGAUGAAATT-3'
	Antisense	5'-UUUCAUCAGCAGCAUAUCCTT-3'
mALKBH5 siRNA-1	Sense	5'-GAGAACUAUUGGCGCAAAUTT-3'
	Antisense	5'−AUUUGCGCCAAUAGUUCUCTT-3'
mALKBH5 siRNA-2	Sense	5'-CUGCGCAACAAGUACUUCUTT-3'
	Antisense	5'-AGAAGUACUUGUUGCGCAGTT-3'
mALKBH5 siRNA-3	Sense	5'-GCGACUACGAGGAGCAUCATT-3'
	Antisense	5'-UGAUGCUCCUCGUAGUCGCTT-3'
mYTHDF1 siRNA	Sense	5'-GAUCCUUACCUGUCCAGUUACTT-3'
	Antisense	5'-GUAACUGGACAGGUAAGGAUCTT-3'
mYTHDF2 siRNA	Sense	5'- GUAACUGGACAGGUAAGGAUCTT-3'
	Antisense	5'-UAUAUCCAGAGCUUUGAGUTT-3'

Table S4. Cardiac function measured by echocardiography.

Cardiac function measured by echocardiography of WT and ALKBH5 KO mice.

	**WT**	**ALKBH5-KO**
**IVSd (mm)**	0.89±0.02	0.98±0.08
**IVSs (mm)**	1.28±0.10	1.45±0.09
**LVIDd (mm)**	2.08±0.20	2.08±0.21
**LVIDs (mm)**	0.88±0.09	0.98±0.13
**LVPWd (mm)**	1.13±0.19	1.08±0.14
**LVPWs (mm)**	1.57±0.20	1.48±0.14
**EF (%)**	89.13±1.72	85.11±3.11
**FS (%)**	57.45±2.88	52.74±3.73
